# Three-dimensional AlZnO/Al_2_O_3_/AlZnO nanocapacitor arrays on Si substrate for energy storage

**DOI:** 10.1186/1556-276X-7-544

**Published:** 2012-10-02

**Authors:** Lian-Jie Li, Bao Zhu, Shi-Jin Ding, Hong-Liang Lu, Qing-Qing Sun, Anquan Jiang, David Wei Zhang, Chunxiang Zhu

**Affiliations:** 1 State Key Laboratory of ASIC and System, School of Microelectronics, Fudan University, Shanghai 200433, People's Republic of China; 2 Silicon Nano Device Laboratory, Department of Electrical and Computer Engineering, National University of Singapore, Singapore 119260, Singapore

**Keywords:** Nanocapacitor arrays, AAO, Capacitance density, RC time constant, Energy storage

## Abstract

High density three-dimensional AZO/Al_2_O_3_/AZO nanocapacitor arrays have been fabricated for energy storage applications. Using atomic layer deposition technique, the stack of AZO/Al_2_O_3_/AZO has been grown in the porous anodic alumina template which is directly formed on the Si substrate. The fabricated capacitor shows a high capacitance density of 15.3 fF/μm^2 ^at 100 kHz, which is nearly 2.5 times over the planar capacitor under identical conditions in theory. Further, the charge-discharge characteristics of the capacitor are characterized, indicating that the resistance-capacitance time constants are equal to 300 ns for the charging and discharging processes, and have no dependence on the voltage supply. This reflects good power characteristics of the electrostatic capacitor.

## Background

In recent years, the novel characteristics of nanostructures have attracted great attention from researchers; in particular, the nanostructure-based devices have been explored in many fields such as electronics, optoelectronics, and magnetism [1-3]. As one of the most important applications, the nanocapacitor arrays have been intensively studied for the next generation energy storage system due to increasing demands of high capacity, lightweight, and compact energy storage devices [4-7].

In terms of the electrostatic capacitor theory, the capacitor capacity is mainly determined by the electrode area. Therefore, to increase the effective area of electrodes, the three-dimensional nanocapacitor arrays are introduced to achieve a high capacitance density. As one of the most promising methods of fabricating nanocapacitor arrays, the porous nanostructure templates are used widely, including nanowire, nanopillar, anodic alumina (AAO), and so on [4-7]. For example, although InAs nanowire-based nanocapacitors (Au/Cr/HfO_2_/InAs) can achieve a larger electrode surface area, the poor mechanical strength of nanowires makes it unsuitable for energy storage [4]. Moreover, a significant improvement in capacitance has been achieved for the template of silicon nanopillars, which was fabricated by Au metal-assisted etching in conjunction with interference lithography; however, the Au residue could cause oxide degradation and inferior device performance [5]. On the other hand, porous AAO templates made from aluminum foils exhibit a high degree of regularity and uniformity in addition to a quite simple process. Therefore, the performance of the fabricated nanocapacitors has been improved significantly [6,7]. However, it is hard to transfer the AAO template onto other substrates (e.g., Si substrate) due to its fragileness [8]. Although Banerjee et al. reported that the aluminum foils were first bonded anodically to the glass substrate and then AAO template was formed by two steps of anodization [7], it faces a complex processing. Therefore, it is expected that the AAO template can be formed directly on the Si substrate without template transfer or complex bonding.

On the other hand, the electrode material of the capacitor also plays an important role in the performance of nanocapacitor arrays. As a transparent conductive oxide material in optoelectronics, Al-doped ZnO (AZO) has many attractive characteristics including excellent thermal stability, low resistivity, low manufacture cost, and so on [9]. Therefore, the introduction of AZO as the electrode of nanocapacitor arrays could boost the energy storage device integrated with the optoelectronic device.

In this study, we demonstrate the successful fabrication of AZO/Al_2_O_3_/AZO nanocapacitor arrays in the porous AAO template, which is directly formed on Si substrate by two-step anodization. The resulting nanocapacitor arrays show a high capacitance density of 15.3 fF/μm^2^, which is nearly 2.5 times that of the planar capacitor. Furthermore, the charge-discharge characteristics of the nanocapacitor arrays are also discussed.

## Methods

**Figure 1:**
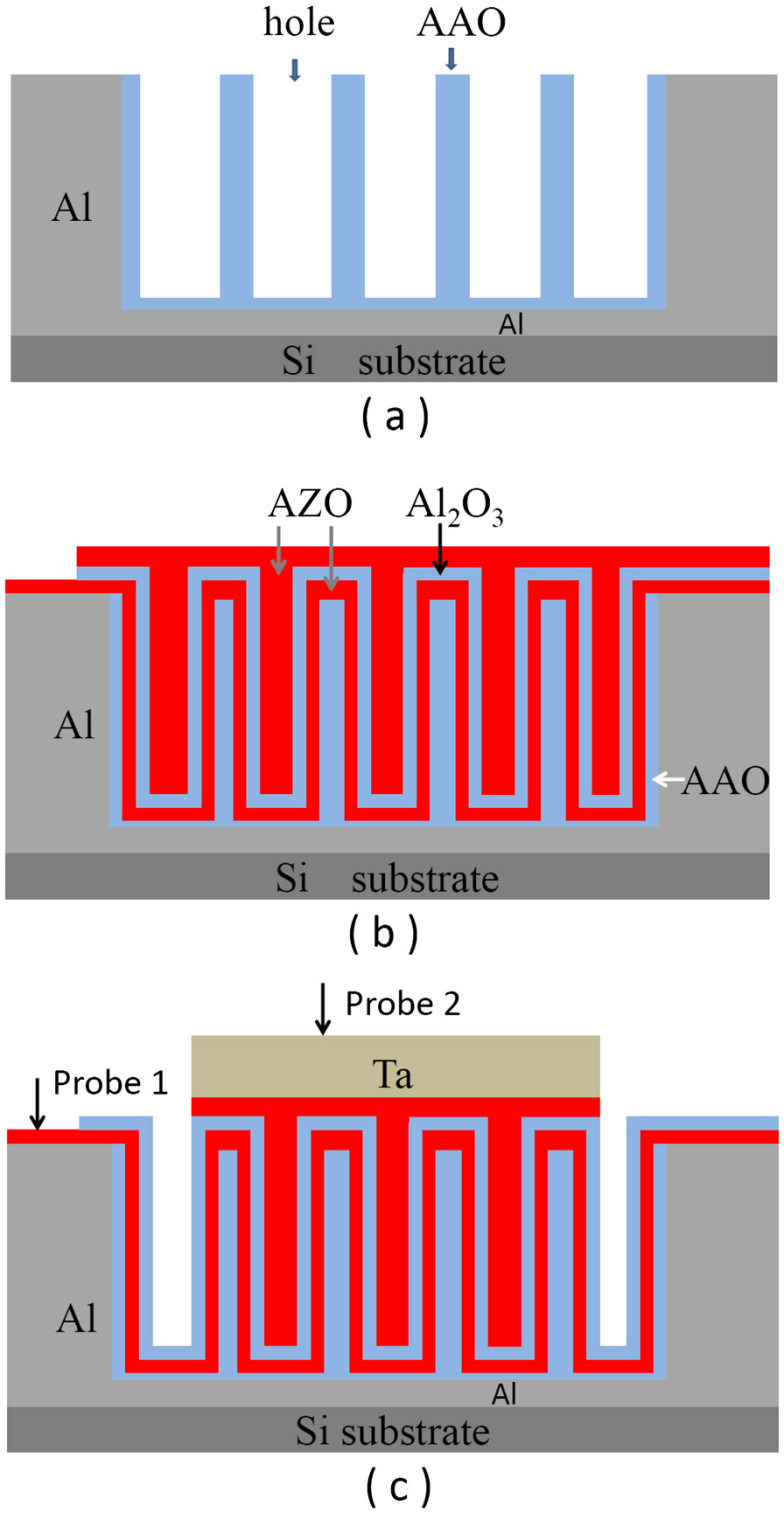
**Schematic diagrams of fabrication processes for three-dimensional AZO/Al**_**2**_**O**_**3**_**/AZO nanocapacitor arrays.** (**a**) The formation of AAO templates on the Si substrate, (**b**) atomic layer deposition of the AZO/Al_2_O_3_/AZO stack (defined as MIM structure), and (**c**) the formation of capacitors for electrical measurements, including three-dimensional nanocapacitor arrays and a top contact layer of Ta film.

The fabrication steps of the AZO/Al_2_O_3_/AZO nanocapacitor arrays are illustrated schematically in Figure 1. Firstly, an aluminum film with a thickness of 1 μm was deposited on the cleaned Si substrate by thermal evaporation, which was followed by annealing in N_2 _at 500°C for 2 min in order to enhance the adhesion between the Al film and the Si substrate. The porous AAO was then formed by two steps of anodization, i.e., the anodization of the aluminum film was carried out in a 0.3-M oxalic acid solution at 25°C under 40 V, then the pore widening was performed in a 5-wt.% phosphoric acid solution. As a result, the resulting AAO template has uniform nanopores with a diameter of approximately 80 nm and a density of approximately 1 × 10^10 ^cm^−2^. Subsequently, the stack of AZO/Al_2_O_3_/AZO (12/10/12 nm) was deposited in the AAO template by atomic layer deposition (ALD). Herein, the AZO films were used as both bottom and top electrode plates and were composed of alternate 20 cycles of ZnO and 1 cycle of Al_2_O_3_. ZnO and Al_2_O_3 _were grown from the precursors of diethyl zinc/H_2_O, and trimethyl aluminum/H_2_O at 200°C, respectively. Whereafter, the top Ta electrode with a thickness of 180 nm was deposited on the stack through a hard mask by magnetron sputtering. Subsequently, the top AZO film outside the Ta electrodes was etched by 0.02 wt.% HCl aqueous solution; thus, the top electrodes of AZO/Ta were formed. It is worthwhile to point out that only the central round part of the Al film on the Si substrate was anodized, so the fringe part of the Al film is preserved to serve as the bottom electrode. After deposition of the bottom AZO layer, a small region within the fringe part of Al film was protected from subsequent ALD deposition and chemical etching process, which is used as the probe position during electrical measurements. Accordingly, the capacitors consisting of nanocapacitor arrays were formed for electrical characterization. The *C*-*V* characteristic of the nanocapacitor arrays was measured using an Agilent 4294A precision LCR meter, and the charge-discharge characteristics were measured using Agilent 33250A (Agilent Technologies, Inc., Germany).

## Results and discussion

**Figure 2:**
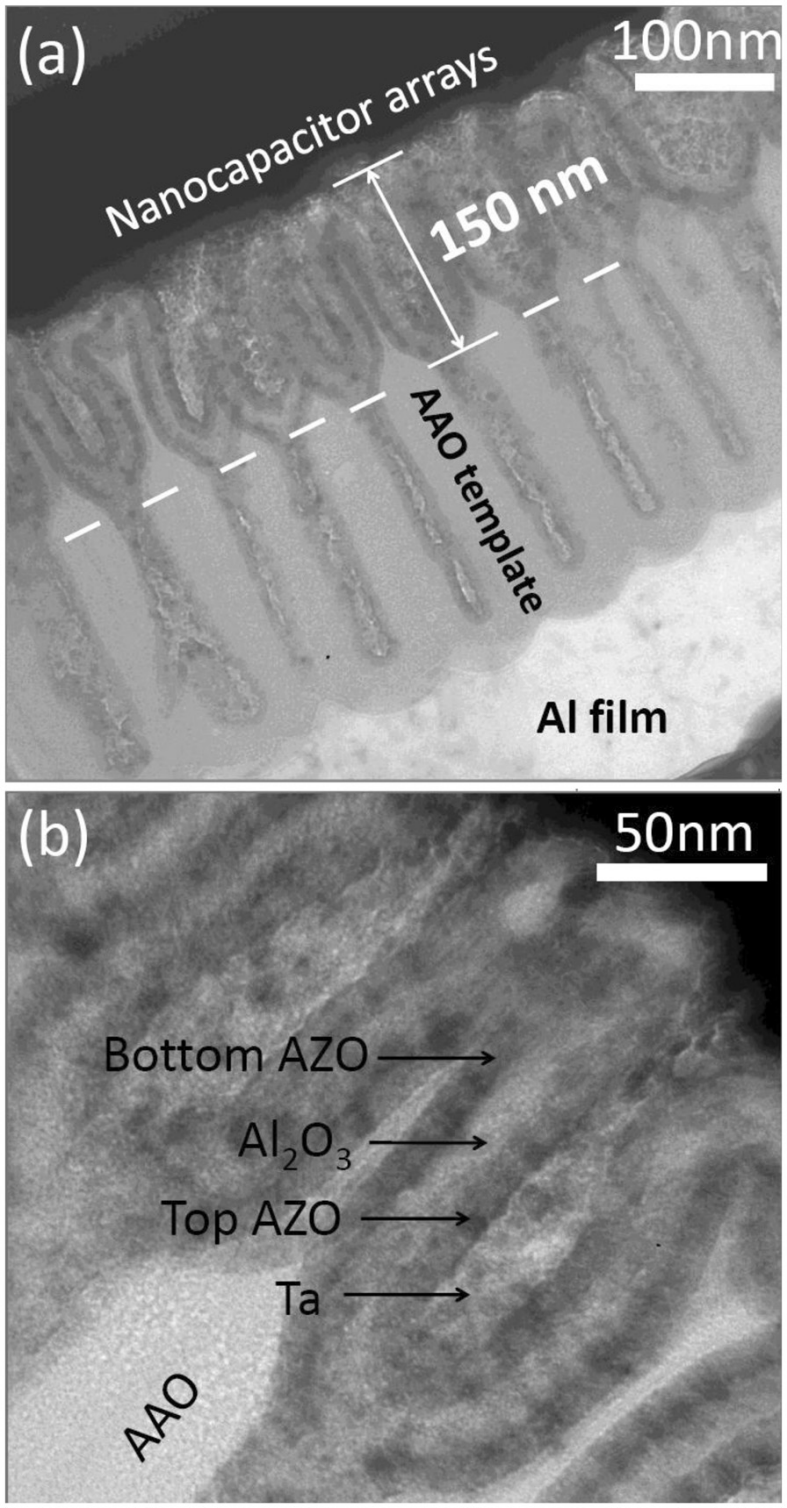
**Cross-sectional TEM images of (a) nanocapacitor arrays embedded into AAO template and (b) three-dimensional AZO/Al**_**2**_**O**_**3**_**/AZO stack.**

Figure 2 shows the cross-sectional transmission electron microscopy (TEM) images of the fabricated nanocapacitor arrays. It is found that the fabricated three-dimensional nanocapacitor arrays are embedded into the AAO template. Under the AAO template, a layer of Al is observed clearly, which is used to maintain good contact between the AAO and the Si substrate. This can be realized by partial anodization of the aluminum film. In our experiment, the depth of the formed pores is close to 400 nm, which can be adjusted through the thickness of the initial Al film and the time of anodization. Furthermore, it is interesting to find that each pore includes the top wide part and the bottom narrow part; the former exhibits a diameter of approximately 80 nm and a depth of approximately 150 nm, and the latter exhibits a diameter of approximately 20 nm and a depth of approximately 250 nm. As the pore diameter is proportional to the anodic voltage, the above-mentioned phenomenon could be attributed to the voltage fluctuation during the anodization process [10]. It is worth mentioning that the bottom holes can be filled fully by AZO after the deposition of a 12-nm AZO film due to their small diameters; therefore, the bottom part does not contribute a lot to the capacitance density because the surface area of the bottom electrode does not increase further. Figure 2b shows the high-resolution TEM image of the nanocapacitor. It is found that the insulator of Al_2_O_3 _is sandwiched well by the AZO films, and no void can be seen in the nanopores. 

**Figure 3:**
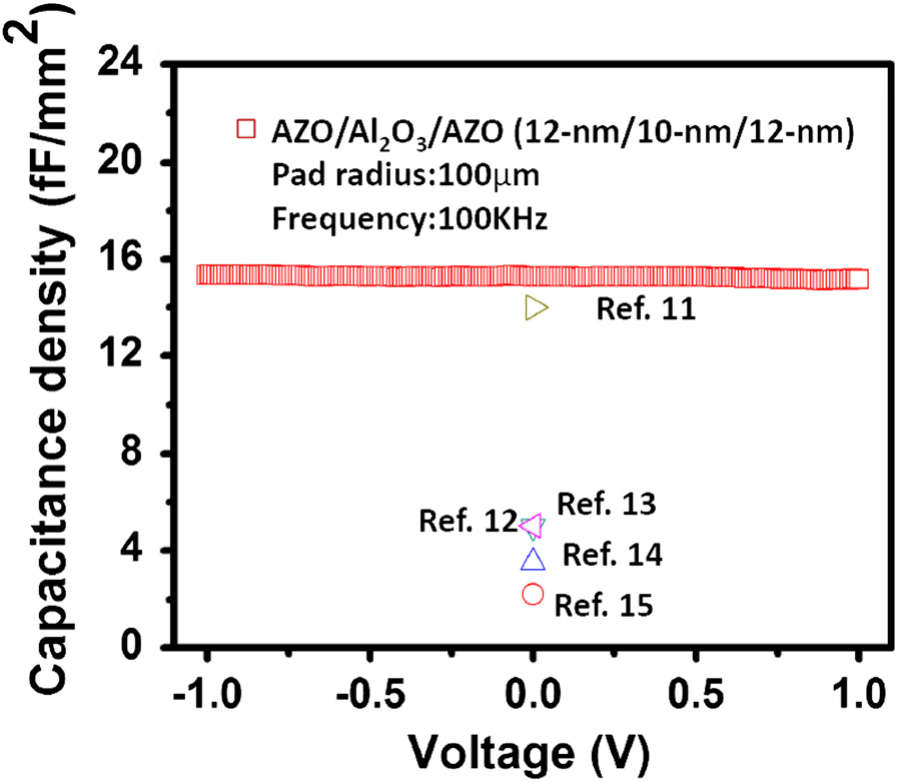
**The *C*-*V *characteristic of the fabricated capacitor with three-dimensional nanocapacitor arrays.** It is compared to other reported planar MIM capacitors with Al_2_O_3 _dielectrics.

Figure 3 shows the typical *CV* curve of the fabricated capacitor with the AZO/Al_2_O_3_/AZO nanocapacitor arrays at 100 kHz. The extracted capacitance density reaches 15.3 fF/μm^2^, which is nearly 2.5 times that of the planar capacitor under identical conditions. By comparison with the planar Al_2_O_3 _dielectric MIM capacitors [11-15], the fabricated capacitor in this study exhibits a significant increase in capacitance density, as shown in Figure 3. Although another study [11] also demonstrates a comparable capacitance density, this is due to a very thin Al_2_O_3 _film of 5 nm. Further, the total capacitance of the nanocapacitor structure can be calculated according to Equations 1, 2, 3, and 4 [7]:

(1) $$C_{\text{total}}=a\left(C_{\text{planar}}+C_{\text{pore}+}C_{\text{bottom}}\right)$$

(2) $$C_{\text{planar}}=\left(\frac{k{ɛ}_{0}}{t_{\text{insulator}}}\right)\left[\sqrt{\frac{3}{2}\times {\left(2r_{\text{pore}}+D\right)}^{2}-\pi r_{\text{pore}}^{2}}\right]$$

(3) $$C_{\text{bottom}}={ɛ}_{0}\pi \frac{{\left[r_{\text{pore}}-\left(t_{\mathrm{BE}}+t_{\text{insulator}}\right)\right]}^{2}}{t_{\text{insulator}}}$$

(4) $$C_{\text{pore}}=\frac{2\pi k{ɛ}_{0}L}{\ln\left[\frac{\left(r_{\text{pore}}-t_{\mathrm{BE}}\right)}{r_{\text{pore}}-\left(t_{\mathrm{BE}}+t_{\text{insulator}}\right)}\right]}$$

Here, *α* represents the density of pores, which is close to 1 × 10^10^ cm^−2 ^in the present experiment according to the scanning electron microscope image of the AAO template (not shown here); *k* is the dielectric constant of Al_2_O_3_ (*k* = 7.6), and *t*_BE_ and *t*_TE_ correspond to the thicknesses of the bottom AZO layer and the top AZO layer, respectively, i.e., 12 nm. The depth (*L*) and radius (*r*_pore_) of nanopores are approximately 150 and 40 nm, respectively. *D* represents the interpore distance, which is 10 nm. Therefore, the calculated *C*_planar_, *C*_bottom_, and *C*_pore _are equal to 1.3 × 10^−2^, 0.068 × 10^−2^, and 14.3 × 10^−2^ fF, respectively; thus, the total capacitance density (*C*_total_) amounts to 15.7 fF/μm^2^, which is close to the measurement result. In addition, this also indicates that *C*_total_ is dominated by *C*_pore_. Further, the capacitance density can be enhanced by increasing the height of nanopores. Moreover, the parameters of *t*_BE_, *r*_pore_, and *t*_insulator _have positive or negative effects on the capacitance density. Therefore, to achieve a maximum capacitance density for practical applications, we have to consider the effects of various parameters, especially the influence of the insulator thickness on the leakage current, in order to make a balance between high capacitance and low leakage current.

On the other hand, although a high capacitance density has been achieved, the leakage current characteristic is not satisfactory (not shown here). This is due to the inner surface roughness and chemical contamination of the template, thus resulting in local high electric fields and degrading the leakage current characteristics. The aforementioned phenomena are also reported by other groups [7,16]. Further, it is reported that the leakage current can be reduced remarkably by the introduction of barrier anodic alumina and/or ALD passivation layers in the AAO template. As an example, the leakage current density can decrease from 1×10^−3^ A/cm^2^ to 1×10^−9^ A/cm^2 ^at 3 MV/cm [16].

**Figure 4:**
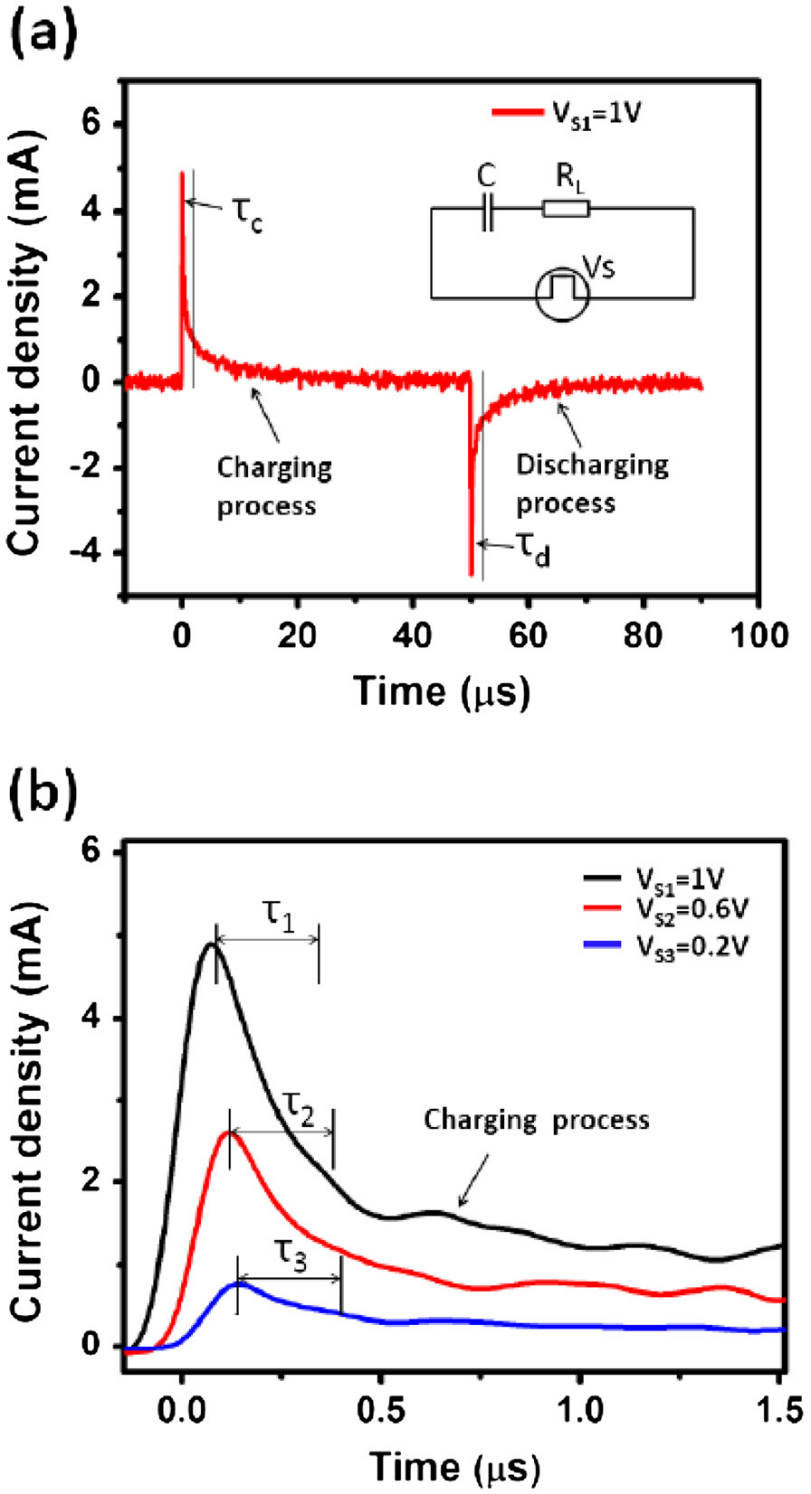
**Charge-discharge curves and charging process of the capacitor.** (**a**) The charge-discharge curves of the fabricated capacitor with an electrode radius of 400 μm, and the inset shows the equivalent resistor-capacitor circuit. (**b**) The charging process of the capacitor under different voltage supplies such as 1, 0.6, and 0.2 V.

According to the charge-discharge process of the resistor-capacitor circuit, the resistance-capacitance (RC) time constant determines the charge-discharge rate of capacitor, i.e., the power characteristics of capacitors [17]. The RC time constant is defined as *τ*, which means the length of time when the circuit current attains *e*^−1^ (i.e., 36.8%) of the initial value. Figure 4a shows the whole charge-discharge process of the nanocapacitor arrays with an electrode radius of 400 μm, and the inset is an equivalent resistor-capacitor circuit. The time constant is characterized by *τ*_c _in charging process as well as *τ*_d _in discharging process. Under voltage supply of 1 V, both *τ*_c _and *τ*_d _are equal to 300 ns, and such short time constants are in good agreement with the power characteristics of electrostatic capacitors. Figure 4b shows dependence of the charging current on time. Under different voltage supplies, i.e., *V*_S1_ = 1 V, *V*_S2_ = 0.6 V, and *V*_S3_ = 0.2 V, the resulting time constants of *τ*_1_, *τ*_2_, and *τ*_3 _are equal to 300 ns for the capacitor with an electrode radius of 400 μm, respectively. This reveals that the time constant has no dependence on the voltage supply, which is in accordance with the RC charge-discharge theory. As a result, the nanocapacitor arrays can meet a high capacitance density without sacrificing the power characteristics of the electrostatic capacitor. 

## Conclusions

In summary, high capacitance density nanocapacitor arrays have been fabricated via porous AAO template directly on silicon substrate and ALD processing. The nanocapacitor arrays based on the stack of AZO/Al_2_O_3_/AZO (12/10/12 nm) exhibit a high capacitance density of 15.3 fF/μm^2^, and the RC time constant is 300 ns, indicating good power characteristics of the electrostatic capacitor. As a result, in combination with flexible electronics and energy transformation components such as solar cells, nanocapacitor arrays could be a promising candidate as energy storage devices.

## Competing interests

The authors declare that they have no competing interests.

## Authors’ contributions

LJL carried out the main part of fabrication and analytical works. BZ and SJD participated in the sequence alignment and drafted the manuscript. HLL, QQS, AQJ, DWZ, and CZ conceived the study and participated in its design. All authors read and approved the final manuscript.
